# Characterizing conflict between humans and big cats *Panthera spp*: A systematic review of research trends and management opportunities

**DOI:** 10.1371/journal.pone.0203877

**Published:** 2018-09-18

**Authors:** Kathleen Krafte Holland, Lincoln R. Larson, Robert B. Powell

**Affiliations:** 1 Dept. of Parks, Recreation & Tourism Management, Clemson University Clemson, SC, United States of America; 2 Dept. of Parks, Recreation & Tourism Management, North Carolina State University, Raleigh, NC, United States of America; Universitat Autonoma de Barcelona, SPAIN

## Abstract

Conservation of big cats (*Panthera* spp.), a taxonomic group including tigers, lions, jaguars, leopards and snow leopards, is a daunting challenge. As expanding human populations across *Panthera* range countries exacerbate competition for land and prey, conflicts between humans and big cats are inevitable. Through a systematic review of the peer-reviewed literature published from 1991 to 2014 and indexed in Web of Science and Google Scholar (186 articles), our study explored the current state of knowledge regarding human-*Panthera* conflict and potential solutions, examining variables such as spatial and temporal distribution of research, methods used to study conflict, evaluation of interventions, and management recommendations. Our synthesis revealed several key data gaps and research needs. More studies could utilize diverse data collection approaches to focus on both the ecological and socio-cultural context for conflict. Additionally, only 21% of articles included in the review evaluated conflict mitigation interventions, and few of these yielded conclusive results. Success ratios suggest that compensation schemes and livestock management strategies were more effective tools for addressing conflict than either direct interventions (lethal removal or translocation of animals) or community interventions (e.g. education, ecotourism, local management). More studies should systematically evaluate the efficacy of conflict mitigation strategies, many of which are consistently recommended without empirical support. Results highlight trends and opportunities that can be used to inform future research and management efforts focused on human-*Panthera* conflict, ultimately enhancing the potential for coexistence between humans and carnivore species worldwide.

## Introduction

Big cats (*Panthera* spp.), a taxonomic group that includes tigers, lions, jaguars, leopards, and snow leopards, are apex carnivore species that drive the structure and function of biological communities in diverse ecosystems around the world [1]. These majestic creatures have also been a source of apprehension, intrigue, and inspiration throughout human history [2]. Consequently, big cat conservation has emerged as an important global priority, yet one that remains a daunting challenge. According to the IUCN Red List, tigers (*Panthera tigris*) are classified as ‘endangered’ with a population of 3,200, lions (*Panthera leo*) are classified as ‘vulnerable’ with worldwide populations <30,000, jaguars (*Panthera onca*) are classified as ‘near threatened’ with worldwide populations of about 18,000, and leopards *(Panthera pardus)* are classified as ‘near threatened’ with worldwide populations unknown [3].

As keystone species in their ecosystems, these predators are essential to maintaining biodiversity and ecosystem balance [4]. Because big cats require large territories and plentiful prey populations to survive, conservation efforts aimed at preserving these species have the potential to produce significant biodiversity gains across multiple taxa [5]. However, expanding human populations and development have exacerbated competition for land and prey between people and big cats in *Panthera* range countries, inevitably producing conflict [6–8]. Human-wildlife conflict is defined as conflict that occurs when the “needs and behavior of wildlife impact negatively on the goals of humans, or when the goals of humans negatively impact the needs of wildlife” [9]. Habitat loss due to land encroachment by humans [10, 11], competition for limited resources such as prey or water [12, 13], and reintroductions of *Panthera* species [14] are all documented sources of conflict between humans and big cats. In many cases, such conflicts result in loss of livestock [15–17] or injury and death to humans [8, 18] and wild animals [19]. Conflict also arises when conservation and human development goals do not align [20, 21], generating disagreements between humans *about* wildlife and conservation priorities [22]. Such conflict may include disputes over protected area boundaries, compensation plans, legal responses to incidents, or injury and death to *Panthera* species [23].

For decades, researchers have employed different disciplinary paradigms and frameworks in an attempt to understand sources of human-wildlife conflict and to identify potential mitigation strategies [24–26]. In many cases, conflict reduction interventions are designed to physically separate big cats and humans, incorporating strategies such as improved livestock husbandry strategies [17, 25, 27], relocation of problem animals [28, 29] or people [30], and killing of problem animals [31–33]. In other cases, interventions have focused more directly on the social, economic, and political factors that fuel conservation-related conflict [23, 24, 34], ranging from financial compensation schemes for predator induced losses [35, 36] to approaches centered on education [37] and sustainable community development [38, 39]. However, despite diligent efforts by researchers, governments, NGOs, and local communities to address conflict and increase tolerance and acceptance capacity for large predators around the world [40, 41], management interventions have achieved limited success [6, 25, 26, 42].

Enhanced sharing of information across disciplines and geographies could help to resolve this complex problem. For example, although many studies have examined different aspects of the contentious relationship between humans and big cats, few have attempted to describe lessons learned from multiple social and ecological perspectives across space and time [34]. Through a review of peer-reviewed literature, our study explores the current state of knowledge regarding human-*Panthera* conflict and potential mitigation strategies to inform future management decisions and research agendas. Our review focused on the five big cats (genus *Panthera*) whose level of conflict with humans has been rated as high (jaguar, snow leopard) or severe (tiger, lion, leopard) [7]. We sought to answer two primary questions: (1) What are the key trends and patterns in human-*Panthera* conflict research?; and (2) Which human-*Panthera* conflict mitigation strategies have proven to be most effective?

## Methods

### Selection of articles

To answer these questions, we searched peer-reviewed articles addressing human-*Panthera* conflict in two comprehensive databases of scientific publications (Web of Science and Google Scholar) in February 2015. To be included in the review, a journal article’s title or key words had to contain at least one of the five *Panthera* species names (or common names) *and* at least one of the following words or phrases: attack, attitude, coexistence, human-wildlife conflict, or livestock (see S1 File). These key words were strategically selected after reviewing a subset of articles on the topic. All results from Web of Science were included in the review, as well as the first 100 results from Google Scholar. Due to the number of articles returned using Google Scholar searches, a complete screening was not possible. Therefore, relevancy of results for all search combinations were examined and it was determined that inclusion criteria were no longer being met past the first 100 results. Following protocols used in similar review articles [25, 26], we included only English language journals. Non-peer reviewed (“grey”) literature was excluded because (a) there was no consistent means to assess the scientific rigor of these publications and (b) there was no systematic method for retrieving this literature. Overall, these searchers returned 5,632 articles.

**Fig 1:**
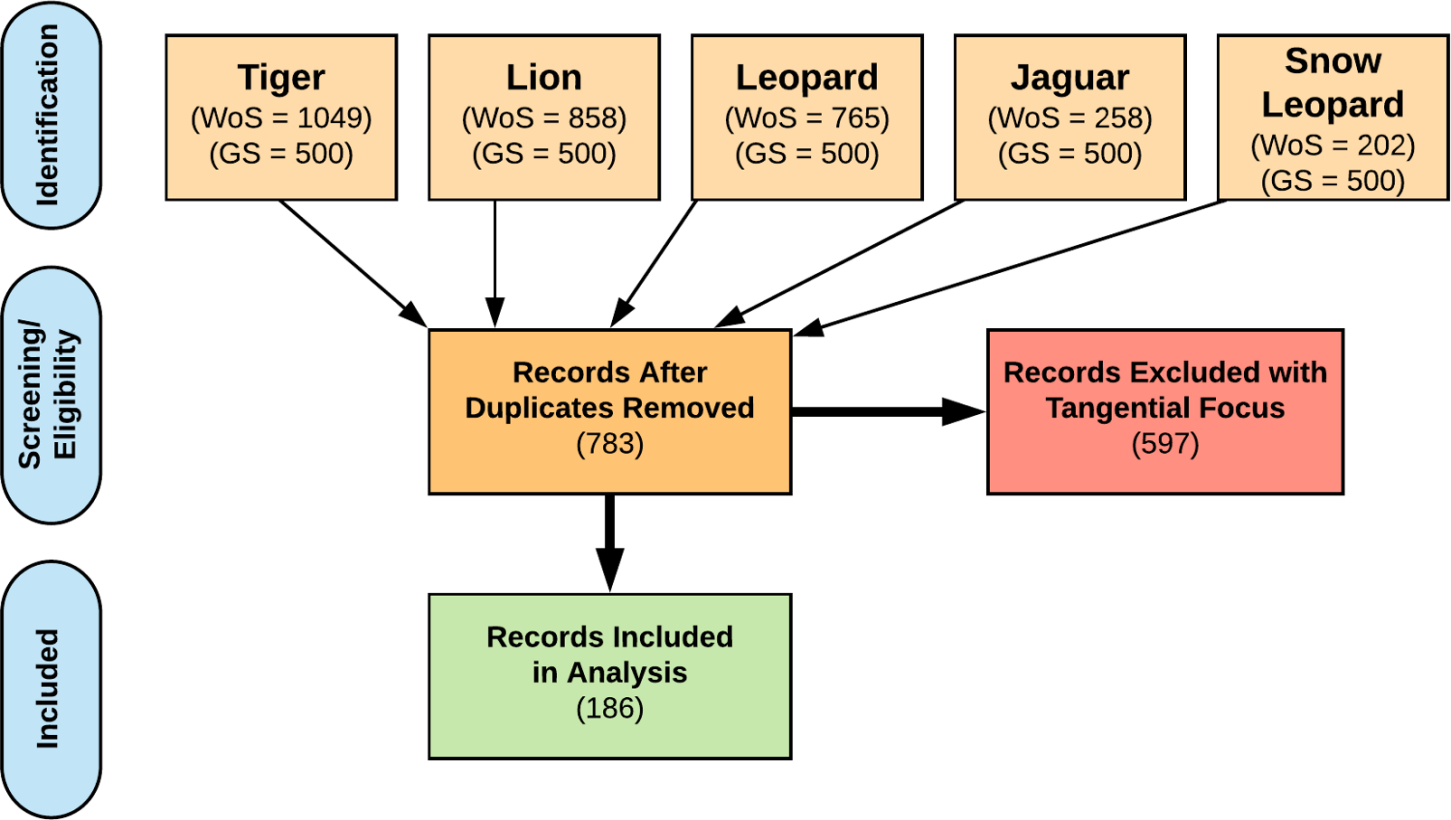
Adapted PRISMA Flow Diagram summarizing total articles found and total articles included in final analysis of human-*Panthera* conflict papers, by species (adapted from Moher et al., 2009). Search engine codes: WoS = Web of Science, GS = Google Scholar. Search terms: each of the five Panthera species names (or common names) and at least one of the following words or phrases: attack, attitude, coexistence, human-wildlife conflict, or livestock. Duplicates included records that appeared multiple times in one search or overlapped between searches. Records were deemed tangential if they focused exclusively on ecological indicators or did not directly assess or evaluate conflict with humans. See S1 File for more details about the literature review search methods and S2 File for a PRISMA reporting checklist.

After removing duplicates from Google Scholar searches and articles that overlapped across multiple searches (additional hits for article across multiple searches), the potential sample was reduced to 783 (Fig 1, see S2 File for PRISMA reporting checklist). Two members of the research team then reviewed the abstracts of selected papers to confirm an appropriate focus on either conflict related to one or more *Panthera* species or broader human-*Panthera* interactions. We excluded articles that (a) did not focus explicitly on at least one *Panthera* species, or (b) did not examine interactions between humans and the focal species. For example, studies with an exclusive ecological focus such as species ranges or prey selection and studies that did not assess or evaluate conflict with humans were removed from the analysis (Fig 1). In total, 186 publications dating from 1991 (earliest article found) to December 2014 (the final search date) were included in the review (Fig 1). To access a full database of articles reviewed, see https://repository.lib.ncsu.edu/handle/1840.20/35459.

### Variable identification and coding

To characterize human-*Panthera* conflict and identify potential mitigation strategies, a random sample of 25 of these 186 articles was selected and screened for variables of interest including location of study, year, publication journal, data collection method, purpose of study, evaluated interventions and recommendations (a proxy for intervention efficacy). A list of specific codes was compiled for topical categories until saturation was reached. Twenty interventions and recommendations that aimed to mitigate human-*Panthera* conflict were identified.

Using content analysis, two researchers then coded a subsample of 25 articles independently without knowledge of each other’s assigned codes following recommendations by Creswell [43] to increase the validity and reliability of results. We then compared coding and reviewed areas of discrepancy until final consensus was reached. All three authors were involved in the coding and discussion of results. Finally, the primary author used the updated coding categories and operational definitions to complete the analysis of the full list of articles. If an article studied more than one *Panthera* species (most commonly involving leopards due to range overlap), the data from that article were included in results for both (or all, if more than two) species. In addition, some relevant studies of human-*Panthera* conflict that were not species-specific (i.e. literature reviews) were also included in the review.

We coded each of the articles for the following general categories (see S3 File for more details about coding interventions):

- *Background Variables*: What was the context in which the study occurred (e.g., continent, country, species)?
- *Purpose of Study*: How did the author(s) define the purpose of their study? The purpose of the article and type of conflict being studied were coded based on the purpose stated by the author(s) (e.g., assess extent of conflict, quantify impact on animals/people, document interventions with or without evaluation).
- *Data Collection Methods*: Were the data collected using social science methods (e.g., data obtained directly from people; interviews, archives, questionnaires), ecological methods (e.g., data not obtained from people; camera trap, observation, field samples, GPS/GIS, radio collars) or a combination of these methods (coded as “hybrid”)?
- *Type of Impact*: What type of impact (e.g., human/animal injury or casualty, impact to human livelihood, livestock loss, ecological impact) was being studied? The type of impact was inferred by the researchers based on the results of each study.
- *Evaluated Interventions*: What conflict mitigation interventions, if any, were evaluated by the authors? Interventions were stated by the author(s) in the methods and/or results sections. Researchers categorized the interventions based on details provided by the author(s) (see S3 File for more details). Interventions included themes such as *livestock management strategies* (dogs, fences, safety gear, night guards, lighting, livestock husbandry techniques, deterring technology, water diversions), *compensation schemes* (proactive or reactive payments), *community interventions* (community conservation/ecotourism, education programs, relocation of people, land management/zoning, legal management, local management, response teams, reporting of incident) and *direct intervention* (hunting of animal, relocation of animal).
- *Recommendations*: What recommended conflict mitigation strategies were ultimately identified by the authors? Recommended interventions to reduce human-*Panthera* conflict were stated by the author(s), usually in the Discussion and/or Conclusion sections, and were based on either (a) the explicit evaluation results reported in the study (if applicable), (b) the expert opinion of the authors, or (c) some combination of the two.

Because indicators of success varied across these interdisciplinary studies and effect sizes were rarely reported, a systematic quantitative comparison of intervention efficacy was not possible. We therefore assessed the efficacy of interventions by calculating subjective success ratios to determine the percentage of articles that both evaluated *and* recommended the same intervention strategy. We assumed that, based on the objective-centered approach frequently used to characterize program success in evaluation research [44] authors would only recommend a strategy they studied if that technique had proven to be effective based on pre-specified parameters. Success ratios for conflict mitigation interventions were therefore estimated using the following general formula:

$$Success\;Ratio=\frac{Number\;of\;articles\;that\;evaluate\;and\;recommend}{Number\;of\;articles\;that\;evaluate}$$

Because the denominator in this ratio only included articles that explicitly evaluated one or more conflict mitigation interventions (n = 39), many articles in our review were omitted from this portion of the analysis.

### Limitations

We encountered several challenges with regards to data collection and coding for this review. We initially intended to assess the causes of human-*Panthera* conflict identified by each study, but this proved to be challenging. For example, habitat loss and resource competition are closely linked to factors such as livelihood structures (i.e., reliance on the natural environment) and environmental policies and practices [45], making causal attributions and coding difficult. The purpose of the articles reviewed was therefore coded based on the stated purpose by the authors in the introduction of the articles. In some cases, stated purposes implied that evaluations of intervention strategies were taking place. However, many of these studies only documented the use of an intervention, not a true assessment of its success in reducing human-wildlife conflict. These studies were therefore omitted from success ratio calculations.

It should also be noted that all articles reviewed were treated as independent studies, even though a few study sites appear to have yielded multiple related articles from the same group of researchers. Additionally, it was difficult to account and control for both social and statistical heterogeneity within our analysis, which integrated studies using various forms of data collection in very diverse research contexts. Assessing the relative quality and/or validity of so many diverse studies was also challenging. By only focusing on published research, our study might also have failed to account for null results, generating a bias toward documentation of positive intervention effects. Because some degree of subjectivity is omnipresent in social science research, we elected to standardize analysis of studies based on how they were conceptualized and conveyed by the authors, not how they were perceived and interpreted by our research team. Overall, we feel that the selection and coding criteria described above allowed for objective analysis of the literature.

Finally, three methodological limitations should be noted. First, our review only includes articles published prior to January 1, 2015. Since that time, the rapidly evolving body of literature on human-wildlife conflict has continued to progress, potentially yielding new insights not extensively reviewed here [25, 26]. Second, our search was confined to English language journals. Although English is widely regarded as the global language of science, this decision may have inadvertently excluded studies published in other non-English journals (e.g., Spanish language journals based in jaguar range countries). Third, although our key words were intentionally selected to identify articles specifically pertaining to human-*Panthera* conflict, these search terms may have inadvertently excluded tangentially related articles such as those focused on community-based natural resource management (e.g., ecotourism initiatives) or other conservation-oriented topics (e.g., trophy hunting, habitat corridor creation). Nevertheless, our search parameters generally paint a comprehensive portrait of the current state of research focused on human-*Panthera* conflict.

## Results

### Research trends and patterns

**Fig 2:**
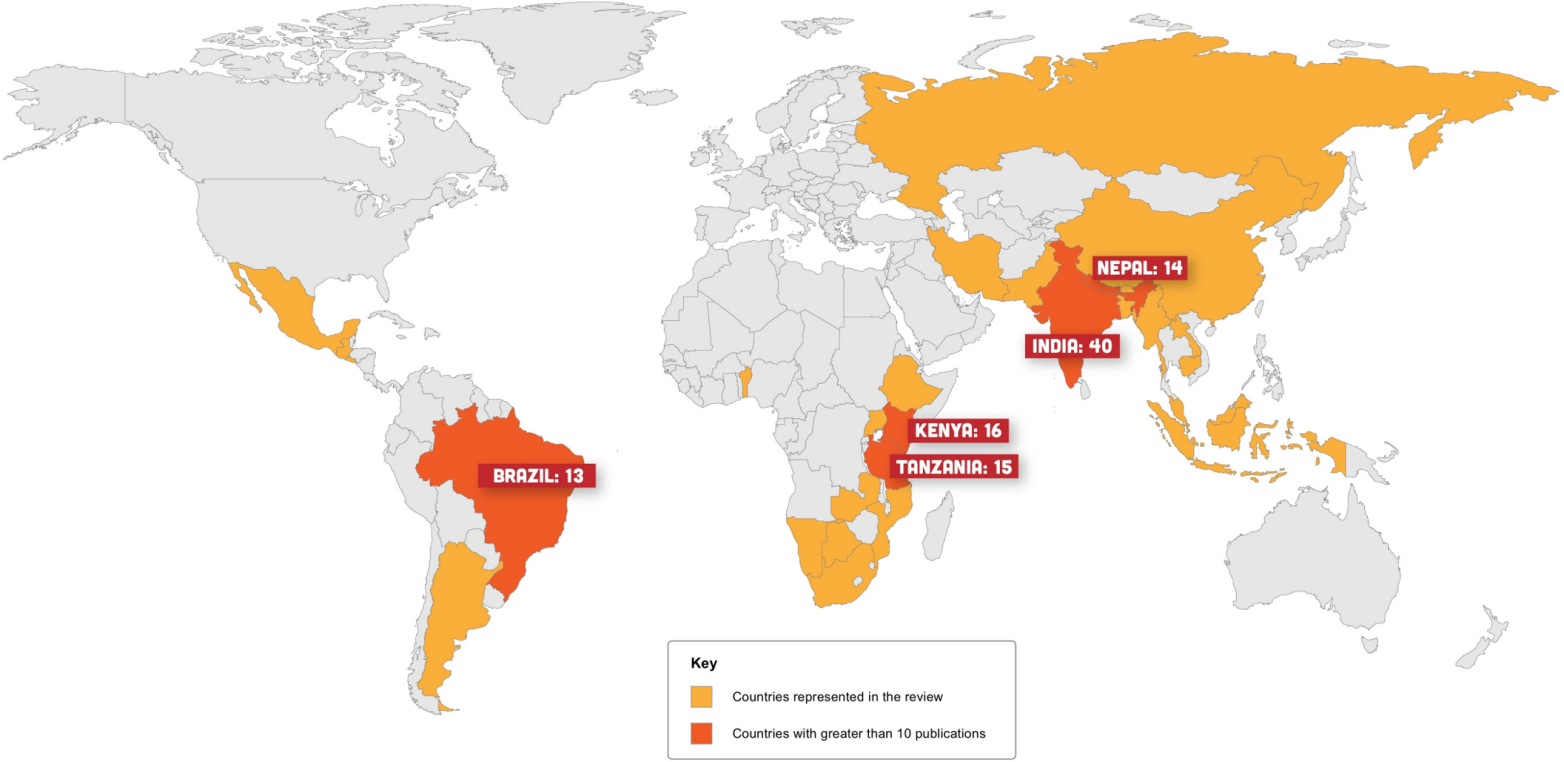
Distribution of research on human-*Panthera* conflict over the past 25 years. All countries that were the focus of at least one study represented in orange; countries that are the focus of more than 10 publications during that period in red. Map created using Adobe Illustrator.

**Fig 3:**
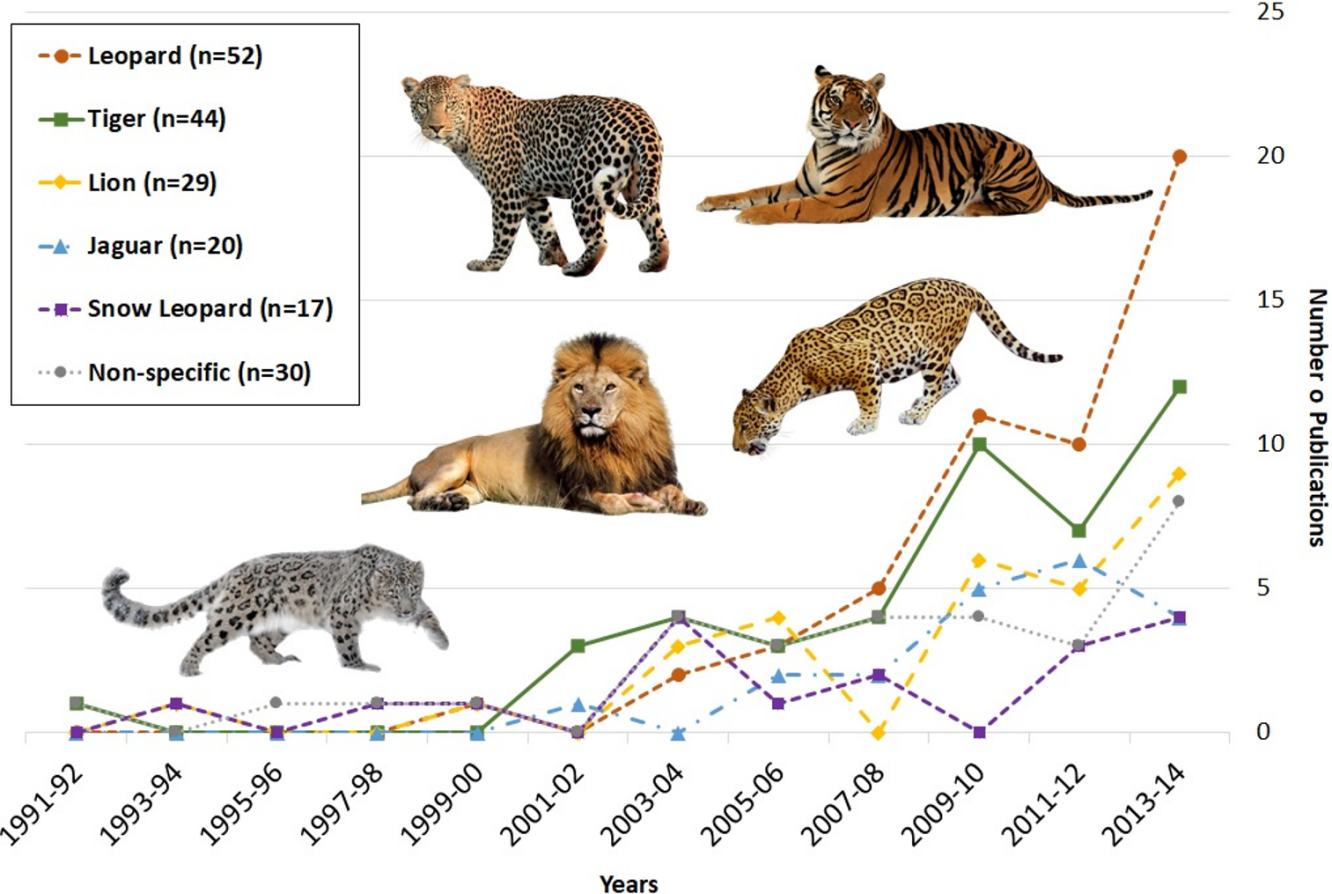
Number of human-*Panthera* conflict peer-reviewed publications over time, by species. Total sample size exceeds the 186 articles reviewed because some articles focused on more than one big cat species. Big cat images adapted and reprinted under a CC BY license.

Our review of human-*Panthera* conflict highlighted study sites in thirty different countries (Fig 2). Distribution of studies generally mirrored species ranges, except for a gap across large portions of jaguar and leopard range. Publication dates for the articles we sampled ranged from 1991 (earliest article identified based on search criteria) to 2014, with the number of published articles increasing over this time period for all five species included in the review (Fig 3). Sixty different peer-reviewed journals were represented in the review.

The author(s) of the articles reviewed reported different reasons for studying human-*Panthera* conflict. The most common purpose (noted in 62 articles) was to simply assess the extent of conflict occurring. As human-*Panthera* conflicts vary in magnitude and severity around the world, it is not surprising that many researchers would aim to characterize the general nature of these interactions. A subset of these articles aimed to quantify the impact of conflict on either humans [33] or wild animals [19], specifically. Other stated purposes included documenting [11] and evaluating interventions [39].

Data collection methods for human-*Panthera* conflict studies varied. Social science research strategies that centered on human responses such as interviews with key stakeholders (63 articles) and archives (67 articles) (e.g., data obtained from news sources, local records) appeared to be the most prevalent form of data collection. Although these social science methods were used for all species, the data collected did not always pertain to socio-cultural themes. For example, interviews and questionnaires were often used to obtain information related to species movements or livestock husbandry techniques, not psychological or cultural factors that might influence conflict. Ecological methods included direct observations of conflict incidents (40 articles) and a variety of tracking and monitoring tools. Radio collars were commonly used for lions whereas camera traps and field samples (e.g. scat) were more common for tigers and leopards. Studies using a combination of ecological and social science data collection methods were rare (29 articles) and were most common for studies focused on snow leopards.

The most commonly studied type of impact was livestock loss, which was addressed by 90 articles. These data are not surprising given the important role that livestock play in the livelihoods of people worldwide, particularly in *Panthera* range countries. Twenty-seven articles examined other impacts to human livelihood such as loss of property or income. These livelihood impacts were most commonly studied with regards to tigers (11 articles), snow leopards (8) and leopards (7). Loss of human life was most often studied with respect to tigers (15). More articles addressed injury or death to *Panthera species* than to humans. This was most common with regards to leopards (14 articles) followed by tigers (12) and lions (10). Only nine articles presented information related to the ecological impact of human-*Panthera* conflicts, and most of these focused on impacts to the prey base.

### Intervention efficacy

Relatively few studies in the sample (n = 39) specifically evaluated conflict mitigation interventions. The most commonly evaluated interventions for almost all species fell into the category of livestock management strategies (34 articles), often focused on physical deterrents such as fences, dogs, and enclosed structures (Table 1). Thirteen articles evaluated compensation schemes and twelve articles evaluated direct interventions. Thirteen articles evaluated community interventions and only four studies evaluated the impact of education programs on human-*Panthera* conflict. Evaluations of interventions involving jaguars were particularly rare.

**Table 1:** Documented efficacy of various intervention strategies to mitigate human-*Panthera* Conflict based on journal articles reviewed from 1991–2014.

Intervention Category (and sub-category)	Evaluate (number of articles)	Evaluate and Recommend (number of articles)	Success Ratio
**Compensation Programs**	**14**	**9**	**0.64**
**Livestock Management Strategies**	**34**	**16**	**0.47**
Livestock Husbandry Techniques	14	10	0.71
Fences	6	3	0.50
Deterrents	6	2	0.33
Dogs	7	1	0.14
Water	1	0	0.00
**Direct Intervention**	**12**	**2**	**0.17**
Hunting of Animal	5	1	0.20
Relocation of Animal	7	1	0.14
**Community Interventions**	**13**	**2**	**0.15**
Community Conservation/Ecotourism	4	1	0.25
Education	4	0	0.00
Local Management	0	0	0.00
Response Teams	3	1	0.33
Land Management/Zoning	1	0	0.00
Relocation of People	1	0	0.00

Although only a small proportion of the articles we examined explicitly evaluated interventions, many of the articles issued specific recommendations for mitigating human-*Panthera* conflict. The most commonly recommended interventions were improved livestock husbandry techniques (e.g. fencing, guard dogs) (54 articles), compensation schemes (44), and education (adult and/or youth outreach) (33). Livestock husbandry was recommended most frequently for lions and leopards whereas compensation schemes and education were recommended more in reference to tiger and snow leopard conflicts. Local management (e.g. community monitoring; 31 articles), and management/zoning (e.g. creation of use/no-use areas; 22) were recommended for all five species. Legal management (e.g. new local or federal laws/regulations; 19) was presented as a recommendation more frequently for tigers than other species. Overall, recommendations encompassed a wide range of interventions–many more than were actually studied in our sample.

Because few studies systematically evaluated specific conflict mitigation interventions, it was difficult to draw definitive conclusions with regards to intervention efficacy. However, based on the evaluation studies that we reviewed, compensation programs and livestock management strategies (fences, dogs, etc.) had the highest success ratios of 0.64 (9 articles evaluating and recommending) and 0.47 (16 articles evaluating and recommending), respectively (Table 1). Successful compensation programs most frequently related to conflicts with snow leopards and tigers, while livestock management tools more commonly related to conflicts with lions. Direct interventions, such as hunting or relocation of problem animals, were less successful (0.17), with only one article evaluating and recommending that approach. Community interventions, which included a wide array of approaches (e.g., ecotourism, education, local management) designed to address and improve the socio-cultural context for conservation, were infrequently evaluated. In the rare cases where such interventions were studied, the estimated success rate was only 0.15 (2 articles evaluation and recommending) (Table 1).

## Discussion

### Trends and patterns in human-*Panthera* conflict research

This review highlights the progress that has been made and the challenges that remain with respect to understanding and addressing human-*Panthera* conflict and the social forces (e.g., policy priorities and practices, research opportunities) that influence it [24, 26]. The number of publications pertaining to human-*Panthera* conflict has increased substantially since the 1990s (Fig 3). As human-*Panthera* interactions and subsequent conflict become more common and conspicuous, making coexistence with carnivores more difficult [6, 46], this trend is likely to continue. Overall, more conflict-related studies have focused on tigers and leopards than other big cat species. These species occur in areas with high human population growth, which may be accelerating the rate of conflict. In addition, these species historically pose more severe threats to humans [37, 47]. The geographical distribution of studies also highlights spatial trends that reflect places experiencing human-*Panthera* conflict and places where researchers are motivated to do something about it (Fig 2). For example, the area with the most published research is India. Not only is India one of the world’s most populous countries, but is also home to leopards and snow leopards, a small population of Asian lions, as well as the highest population of tigers in the world [48]. India also features the social capital, technical resources, and research infrastructure for supporting scientific endeavors. Given the convergence of all of these factors, one might expect India to be an epicenter of big cat conflict research. On the other hand, despite a few recent exceptions [49–51], conflict in the critical jaguar corridor [52] appears particularly under-studied. Future research is needed to fill geographical gaps in current understanding of conflict, particularly in Central America and certain parts of Africa and Southeast Asia where many big cat populations are threatened or endangered.

There are many ecological (e.g., trophic cascades, competition for resources) and sociocultural dimensions (e.g., cultural values, economic resilience) that contribute to the frequency and severity of conflicts in complex social-ecological systems [23, 53], requiring different approaches to data collection. Our review showed that a wide variety of methods have been employed to study human-*Panthera* conflict. Social science methods (interviews, questionnaires, and analysis of archives) were commonly used in the articles included in this study, but the information being gathered often focused on tangible metrics (e.g., frequency of livestock loss, types of predators involved) and rarely accounted for underlying values, attitudes, and norms that may be driving behaviors [40]. For example, Fitzherbert et al. [54] identified collective action and social factors that influenced community support for lion killing in Tanzania, and growing evidence highlights the need to move beyond purely technical fixes or simple dispute resolutions when addressing human-wildlife conflict [34]. More research aimed at identifying the social, cultural, historical, or political drivers of conflict, including those that focus on the process and relationships influencing approaches to conflict resolution, may prove valuable in addressing human-*Panthera* conflict [24, 26, 45].

A number of studies have also utilized ecological data collection methods to understand patterns of human-*Panthera* conflict. By understanding the movement, prey preferences, health, and ranges of animals, researchers may be better able to predict and ultimately prevent incidents of conflict. Ecological data collection methods used to study human-*Panthera* conflict include GIS/GPS [55], camera traps [56], field samples [57], and radio collars [58]. Some studies–particularly those involving leopards and tigers—are exploring the impacts and effects of human impacts on shifting prey bases and trophic cascades, which may force predators to look for alternate food sources such as people and livestock [1, 59–61]. For all *Panthera* species included in this review, however, there is a significant lack of interdisciplinary research that integrates ecological and social science methods to paint a more complete picture of conflict and its effects on both humans and animals [62]. For example, Constant et al. [63] examine a multi-use land system and the management implications for leopard and human populations, highlighting the complexities of approaching conflict from a social-ecological perspective. Efforts to predict conflict using both social and ecological inputs and spatial modeling approaches would also benefit from this type of synthesis [64, 65].

### Efficacy of human-*Panthera* conflict mitigation strategies

Our review revealed a disconcerting finding with significant implications for big cat conservation practice: a noteworthy discrepancy between the number of conflict mitigation recommendations posited by researchers and the number of those interventions whose efficacy has actually been studied and/or systematically evaluated. Similar trends have been reported in other reviews of human-carnivore conflict [25, 26]. Ideally, recommendations for conservation strategies should be evidence-based and anchored in systematic, unbiased evaluation research. In the studies we reviewed, however, this was rarely the case. For example, livestock husbandry was recommended by 45 total articles, yet only 14 articles actually examined specific techniques that could be employed or provided sources or data to document the success of husbandry-related strategies. Similarly, conservation education was recommended in 32 total articles, but education program efficacy was only evaluated in 4 studies.

Overall, four categories of conflict mitigation strategies emerged through the review, demonstrating mixed results in terms of intervention efficacy. Given the small sample sizes and context-specific nature of intervention success [66], our calculted success ratios (Table 1) should be cautiously interpreted. Nevertheless, they complement similar research [25, 26] and provide an informative snapshot of the state of the science with respect to human-*Panthera* conflict management.

#### Compensation programs

Compensation programs revealed the highest success ratios, and were most commonly studied with respect to snow leopards and tigers. Though they require financial resources that may not always be available, payment schemes that reward local people for conserving wildlife and wildlife habitat or, more commonly, compensate people for livestock loss with the hope of preventing the retaliatory killing of predators, can successfully help to secure coexistence between people and predators. In a review of financial instruments, Dickman et al. [35] found that payments to encourage coexistence have great potential in reducing conflicts but are susceptible to many challenges imposed by unique community contexts. Our review supports these findings. For example, a snow leopard depredation compensation plan in Pakistan whose funding is derived from tourism revenue has been successful, but only when tourism profits are sufficient [67]. Similar plans to offset predator-induced damages in India [68] and Botswana [69] described as successful are also compromised due to processing delays, corruption, and award rates that have not matched market values.

Another common challenge related to compensation plan implementation is that many are developed in relation to protected area boundaries. In reality, instances of conflict often occur outside these boundaries. Verifying conflict incidents and identifying who is responsible for compensating local people for wildlife damage outside protected areas (and across jurisdictions) is critical for the future success of this conflict mitigation strategy. The needs for enhanced communication within compensation programs to increase participation, improve response time, enhance transparency, derive fair compensation rates, and create opportunities for local management are commonly cited in the human-wildlife conflict literature [35], and seem to hold true for *Panthera* cats as well. Our review suggests that compensation plans, though they might not be financially feasible in all contexts, have the potential to minimize retaliatory killings of predators while supporting local livelihoods.

#### Livestock management tools

Livestock management strategies were the second most successful types of intervention we studied. This category includes relocation of livestock or shifting herding patterns, fencing, dogs, water or noise deterrents, and other physical barriers. With limited funding and resources to devote to human-cat conflicts in locations around the world, refinement of livestock husbandry techniques may be among the most financially feasible and effective approaches to conflict mitigation, particularly when considering the prevalence of livestock predation among all big cat species [25]. Evaluations of livestock husbandry techniques were most commonly reported in relation to conflicts with lions, which may stem from the widespread traditional free-range grazing practices and the cultural importance of livestock in many cultures across lion ranges [70]. For example, Kuiper et al. [71] showed that seasonal herding changes impacted the rate of predation by lions in communal lands adjacent to Hwange National Park, Zimbabwe, with lion predation increasing significantly in the late growing (wet) season when herds were furthest from their home enclosures and availability of wild prey was lowest. While seasonal patterns of livestock grazing are not uniform across diverse *Panthera* range countries due to extreme variation in climate and topography, knowledge of temporal shifts in depredation clearly aids the development of successful livestock husbandry techniques [72]. Understanding fluctuations in ecological variables such as seasonality, prey abundance, and predator range shifts can assist herders, ranchers, or farmers in decreasing the probability that their livestock are lost.

Spatial management of livestock herds can also play an important role in limiting attacks and losses to carnivores. Herding near villages or areas of high human activity can limit incidents of conflict [17, 71] and requires very little in terms of technical or human capital (e.g., equipment, personnel). Herd species composition also impacts losses due to predators. In Venezuela, cattle herders suffered more loss to jaguar and puma than similar herds that also included buffalo [72]. While making changes to herd composition is often costly, combinations of multiple species may be beneficial in deterring predators.

The use of dogs has been proven effective in limiting livestock losses to big cats with solitary lifestyles, including jaguars and leopards, as well as other cat species such as cheetah and puma in multiple contexts [73, 74]. Despite this efficacy, financial challenges such as purchasing, feeding, and training dogs remain a barrier to their use in conflict mitigation [75]. Other deterrents such as fences, water barriers, or noise deterrents have also been used in an attempt to mitigate conflict with *Panthera* cats. Hayward and Kerley [76] note that human-animal conflict reduction is a primary benefit of fencing. However, they also highlight other costs unrelated to conflict such as ecological impacts or financial burdens that must be considered prior to developing fences or other enclosures. Solar lighting in villages and near livestock enclosures has also been recommended in order to keep predators away from villages and aid in rapid detection [33].

A focus on livestock management strategies is often the most beneficial, practical, and realistic mitigation method for communities that suffer from conflicts with predators [25]. However, all of the livestock husbandry techniques described above require commitment to maintaining and evaluating practices over time. Additionally, focusing exclusively on livestock husbandry for conflict mitigation primarily helps to address issues linked to livestock depredation, and may not be beneficial to communities dealing with *Panthera* attacks on humans or other types of conflict.

#### Direct interventions

Efforts to address conflict by removing problem animals either by hunting, retaliatory killing, or relocation, appeared to achieve little success. Hunting was recommended most frequently for lions, possibly because of their unique appeal to conservation-oriented trophy hunters [77] or the historical role of hunting in many African cultures [70]. However, significant ecological impacts, such as changes in individual territories and impacts to prey species, are often byproducts of lethal control and can exacerbate conflict [78]. Treves [79] noted that the effect of hunting on conflict reduction is unclear and that the assumption hunters will demonstrate stewardship towards carnivores if allowed to hunt them remains unproven. Additionally, because hunters are rarely selective in killing alleged problem animals, other individuals in the population may be inadvertently killed without reducing conflict. In a review of lethal and non-lethal control methods for carnivore conflict with livestock, Treves et al. [32] found insufficient evidence to support the use of lethal control, ultimately recommending that lethal predator control be stopped in instances where significant evidence of functional effectiveness is not available. More research is needed to examine the factors that drive humans to kill carnivores and the impacts of these actions on conflicts and carnivore populations [80].

Direct interventions can also be carried out though translocation of problem animals. However, our review found limited instances where translocation was a success in mitigating conflict. For example, Athreya et al. [28] found that translocation of problem leopards in India led to an increase in conflict and attacks on humans, possibly due to increased aggression stemming translocation stress, movement through unfamiliar human-dominated landscapes, or a decrease in fear or apprehension towards humans. Weise et al. [61] evaluated the efficacy of translocations using Individual Conservation Cost, which is the cost of one successful translocation adjusted by costs of unsuccessful attempts to translocate the same species. Using these calculations, the authors determined that the cost for translocating leopards was too high for both local communities and NGOs to absorb, especially considering the low success rate of many translocation attempts. Collective evidence therefore indicates that, whether problem cats are removed through lethal or non-lethal means, direct interventions are often ineffective and frequently generate more conflict.

#### Community-based interventions

Our review revealed that documented success was also limited for community-based interventions designed to resolve conflict with big cats. These interventions include community-based natural resource management (CBNRM) programs, education and communication initiatives, a focus on local management and monitoring, ecotourism development, or legal management (implementation of policies or enforcement). Such initiatives can benefit communities in many ways [35, 81–83], often by increasing tolerance of communities to predators [41], yet few investigations of CBNRM have focused explicitly on conflict mitigation [84]. Community or stakeholder-based efforts were not well represented in our review of human-*Panthera* conflict or reviews of broader human-wildlife conflict [26] either.

Some research suggests that strategies designed to influence social interactions and cultural cognitions, including social marketing techniques [33] and education [85], can improve communication of costs and benefits that influence tolerance for predators and lead to more positive outcomes for humans and wildlife [34, 46, 49]. Although education was recommended as a community intervention by several authors in our review, none of those studies systematically evaluated education as a conflict mitigation strategy–calling the presumed efficacy of the “cognitive fix” into question.

Local management, which includes community involvement in decision making and strengthening of local leadership in response to conflict (e.g., local response teams), was also highly recommended in our review, but rarely evaluated. Local institutional arrangements are key factors in setting up successful conservation and community programs, but variability in organization and institution structures must be taken into account [23, 81, 86]. In the case of big cats, incidents of conflict could have a particularly significant influence on local livelihoods and community development. In many of the countries studied, big cats represent a major tourism attraction [39, 87]. Revenue from tourism may therefore be an incentive for local communities to invest in conflict mitigation strategies that promote wildlife conservation [88], leading to improvements in quality of life for both people and animals [89]. Interactions between tourists and wildlife have also been the focus of recent research [90, 91], highlighting links between tourism, local communities, and local ecosystems that could positively impact both human livelihoods and big predator conservation efforts. Our systematic review found little evidence to support these claims with respect to big cats, however.

It should be noted that some successful elements of community interventions might have been inadvertently overlooked in our study due to the conflict-centered search terms. For example, specific components of social capital such as reciprocity, social networks, and stakeholder collaboration have been identified as critical to community actions to support or oppose tiger conservation outcomes in India [92] and Malaysia [93]. Though not directly related to conflict mitigation, these studies complement a growing body of literature highlighting potential benefits of conservation (and conflict mitigation) strategies that integrate social, cultural, and historical inputs [24, 26, 34, 45]. Collectively, our results emphasize the need for future research that evaluates different types of community-level interventions and their impact on human-*Panthera* conflict and tolerance for predators.

## Conclusions

This review suggests that, despite a rapid increase in research investigating human conflicts with big cats, many unanswered questions and opportunities remain. Some questions related to human-predator conflict have been addressed in studies published after our literature review was completed in December 2014 [25, 26, 34]. Despite progress, there remains an urgent need for an expanded research agenda to address factors that impact the survival of big cats and the well-being of the people who share their habitat. Improved understanding of conflicts that exist, the reasons they exist, and the efficacy of potential mitigation strategies across diverse settings will help inform future management decisions and promote adaptive responses. Particular emphasis could be placed on collecting data related to individual-level psychological variables that influence wildlife tolerance [40] and community-level cultural and political forces that affect conservation outcomes [34, 45, 66, 92]. A focus on these relationship and process-oriented factors could transform incident-centered conflict resolution paradigms and potentially generate long-term change [24].

Finally, our review echoes previous work and confirms that limited empirical evidence exists to inform recommendations for reducing human-wildlife conflict [25, 26]–and particularly human-*Panthera* conflict [7]—across diverse contexts. While many different big cat conflict mitigation interventions are being recommended and employed across the world, little peer-reviewed information is available to illuminate their effectiveness. The studies that exist suggest that strategies centered on compensation schemes or livestock management practices hold promise for resolving human-*Panthera* conflict, particularly when compared to alternatives such as direct (or lethal) and community-based interventions. Because so few studies have formally examined these strategies, however, it is difficult to draw definitive conclusions and identify best practices. Interventions must be both documented and systematically evaluated over the short and long-term to determine if they are or exacerbating or reducing conflict, ultimately impacting populations of humans and big cats. This is particularly true for community-based interventions such as education, community-based natural resource management, and legal management (policy enforcement and capacity), which are frequently recommended but rarely evaluated. As greater conflict mitigation emphasis is placed on promoting equity and sound governance in addition to technical fixes, investigations of community-based interventions will become even more important [34].

Our global assessment of research trends and opportunities reveals many insights that could be used to inform decisions, management plans, and future projects designed to address human-*Panthera* conflicts, complementing research on human-wildlife conflict involving a broader array of taxa [25, 94]. Local variability involving certain species and ecological, social, or political forces may necessitate different priorities and actions [9]. With a pressing need for conflict resolution and technological advances that facilitate data collection across local and global scales, there is growing hope for big cat conservation. If these efforts are successful (i.e., if big cat populations grow), the potential for conflict will continue to increase [95]. The need for conflict mitigation is therefore inescapable; not only do well-informed interventions have the potential to save iconic carnivore species from extinction, but they also have the potential to foster coexistence by supporting human livelihoods and greater ecosystem health [96, 97]. This review outlines a trajectory for future research focused on human-*Panthera* conflict that may help multiple stakeholders achieve these goals.

## Supporting information

S1 File Literature review search methods. (DOCX)

S2 File PRISMA checklist. (DOCX)

S3 File Definitions and examples of interventions evaluated and/or recommended by articles included in the review. (DOCX)
