# Supplementary material for: Characterizing conflict between humans and big cats Panthera spp: A systematic review of research trends and management opportunities
Source: PLoS One. 2018 Sep 18;13(9):e0203877. doi: 10.1371/journal.pone.0203877 (PMC6143230; doi:10.1371/journal.pone.0203877)
Supplement: S1 File — (DOCX) [file pone.0203877.s001.docx]

**S1 Text.**

**Literature Review Search Methods**

We searched peer-reviewed articles addressing human-*Panthera* conflict in two comprehensive databases of scientific publications (Web of Science and Google Scholar). To be included in the review, a journal article’s title or key words had to contain at least one of the five *Panthera* species names (or common names) *and* at least one of the following words or phrases: attack, attitude, coexistence, human-wildlife conflict, or livestock. These key words were strategically selected after reviewing a subset of articles on the topic. Searches included:

*Panthera leo* AND attack

*Panthera leo* AND attitude

*Panthera leo* AND coexistence

*Panthera leo* AND human-wildlife conflict

*Panthera leo* AND livestock

Lion AND attack

Lion AND attitude

Lion AND coexistence

Lion AND human-wildlife conflict

Lion AND livestock

*Panthera tigris* AND attack

*Panthera tigris* AND attitude

*Panthera tigris* AND coexistence

*Panthera tigris* AND human-wildlife conflict

*Panthera tigris* AND livestock

Tiger AND attack

Tiger AND attitude

Tiger AND coexistence

Tiger AND human-wildlife conflict

Tiger AND livestock

*Panthera onca*  AND attack

*Panthera onca* AND attitude

*Panthera onca* AND coexistence

*Panthera onca* AND human-wildlife conflict

*Panthera onca* AND livestock

Jaguar AND attack

Jaguar AND attitude

Jaguar AND coexistence

Jaguar AND human-wildlife conflict

Jaguar AND livestock

*Panthera pardus*  AND attack

*Panthera pardus* AND attitude

*Panthera pardus* AND coexistence

*Panthera pardus* AND human-wildlife conflict

*Panthera pardus* AND livestock

Leopard AND attack

Leopard AND attitude

Leopard AND coexistence

Leopard AND human-wildlife conflict

Leopard AND livestock

*Panthera uncia*  AND attack

*Panthera uncia* AND attitude

*Panthera uncia* AND coexistence

*Panthera uncia* AND human-wildlife conflict

*Panthera uncia* AND livestock

Snow Leopard AND attack

Snow Leopard AND attitude

Snow Leopard AND coexistence

Snow Leopard AND human-wildlife conflict

Snow Leopard AND livestock

The abstracts of all articles identified using these combinations of search criteria in both Web of Science and Google Scholar. All results from Web of Science were included in the review as well as the first 100 results from Google Scholar. Due to the number of articles obtained using Google Scholar, a complete screening was not possible. Therefore, relevancy of results for all search combinations were examined and it was determined that inclusion criteria were no longer being met past the first 100 results. Results were then reviewed to confirm an appropriate focus on either conflict related to one or more *Panthera* species or broader human-*Panthera* interactions. Articles that did not focus on *Panthera* species, or, did not examine interactions between humans and the focal species (e.g. exclusive ecological focus such as species ranges or prey selection) were excluded. Because some degree of bias is inherent in nearly all social science research, we feel confident in the ability of the review process to objectively assess the literature. As a results, we included all relevant studies in the review.

After removing articles that were not directly relevant, 186 publications dating from 1991 (earliest article found) to December 2014 (the final search date) were included in the review. Following protocols used in similar review articles, we included only English language journals. Non-peer reviewed (“grey”) literature was excluded because (a) there was no consistent means to assess the scientific rigor of these publications and (b) there was no systematic method for retrieving this literature.
