# Supplementary material for: Characterizing conflict between humans and big cats Panthera spp: A systematic review of research trends and management opportunities
Source: PLoS One. 2018 Sep 18;13(9):e0203877. doi: 10.1371/journal.pone.0203877 (PMC6143230; doi:10.1371/journal.pone.0203877)
Supplement: S3 File — (DOCX) [file pone.0203877.s003.docx]

**S3 Table.**

**Definitions and Examples of Interventions Evaluated and/or Recommended by Articles Included in the Review** (see corresponding data file for more article details)

|  | | | | |
| --- | --- | --- | --- | --- |
| Term Definition Examples | | | | |
| Compensation | | Monetary payment provided to those who have suffered loss (human casualty/injury or property damage (livestock or other) due to Panthera conflict. Reporting system in place. | | Evaluated; Karanth et al. 2013, Kgathi et al. 2012 Recommended; Ogra and Badola 2008, Lindsey et al. 2013 |
| Community Conservation/Ecotourism | | Conservation programs designed to provide community benefits, such as ecotourism, used to provide incentives to reduce conflict | | Evaluated; Bagchi and Mishra 2005, Bajracharya et al. 2006 Recommended; Lindsey et al. 2013, Hazzah et al. 2009 |
|  | |  | |  |
|  | |  | |  |
|  | |  | |  |
| Education programs | | Targeted education programs designed to teach value of biodiversity and importance of carnivore species in ecosystems as well as tolerance and conflict mitigation strategies | | Evaluated; Balme et al. 2009, Nugraha and Sugardjito 2009 Recommended; Rust and Marker 2013, Dhanwatey et al. 2013 |
|  | |  | |  |
| Fences | | Fences constructed around livestock areas or villages | | Evaluated; Kolowski and Holekamp 2006, Hayward and Kerley 2009 Recommended;  Kissui 2008, Pettigrew et al. 2012 |
| Hunting of animal | | Intentional tracking and killing of Panthera individual(s) | | Evaluated; Carvalho and Pezzuti 2010, Barlow et al. 2010 Recommended; Zimmerman et al. 2005, Stein et al. 2010 |
| Land management/zoning | | Land designated for particular types of use with the intention of reducing conflict occurrence | | Evaluated; Balme et al. 2009, Goodrich 2010 Recommended; Rust and Marker 2013, Johnson et al. 2006 |
| Legal management | | Legal policies should be developed or revised to reduce conflict | | Recommended; Karanth et al. 2013, Carvalho and Pezzuti 2010 |
|  | |  | |  |
| Livestock Management Strategies | | | |  |
| *Dogs* | | Dogs used to alert/deter attacks (dogs also documented be a cause of attack) | | Evaluated; Bauer et al. 2010, Atickem et al. 2010 Recommended; Pettigrew et al. 2012, Weise et al. 2014 |
| *Enclosed structure* | | Enclosed structure to keep livestock in at night to prevent attacks | | Evaluated; Karanth et al. 2012, 2013,  Kolowski and Holekamp 2006 |
| *Distribution of safety gear* | | Helmets, protective gear provided to hunters and other humans going into forests where conflict has been documented | | Evaluated; Barlow et al. 2010 |
| *Deterring technology* | | Noise generators, electricity, firecrackers, etc. used to intimidated animals and keep them from villages | | Evaluated; Tweheyo et al. 2012, Nugraha and Sugardjito 2009 Recommended; Kolowski and Holekamp 2006, Kgathi et al. 2012 |
| *Lighting* | | Lighting around livestock areas and villages to deter animals | | Evaluated; Karanth et al. 2012, 2013 |
| *Livestock management techniques* | | Improvements to livestock husbandry techniques | | Recommended; Michalski et al. 2005, Dickman et al. 2014 |
| *Night guards* | | Human guards to stay with livestock at night to prevent attacks | | Karanth et al. 2012, 2013, |
| *Relocation of livestock* | | Livestock herds relocated in order to reduce conflict occurrence | | Evaluated; Woodroofe et al. 2006 |
| *Water diversions* | | Man-made ponds or channels created to prevent Panthera individuals from reaching villages or herds | | Evaluated; Tweheyo et al. 2012, Barlow et al. 2010 |
| Local management | | Management should be developed and enforced by local governments | | Recommended; Nugraha and Sugardjito 2009, Dickman et al. 2014 |
|  | |  | |  |
| Proactive payment | | Payment provided to villages or individuals as incentive to avoid conflict | | Evaluated; Pettigrew et al. 2012, Goodrich 2010 |
| Prey management | | Policy in place to artificially regulate or control the Panthera prey populations in the region | | Evaluated; Goodrich 2010 |
| Religion | | Religious beliefs and practices should be taken into account and used as incentive to preserve species and limit conflict | | Recommended; Hazzah et al. 2009 |
| Relocation of animal | | Panthera individual caught and relocated by humans | | Evaluated; Weise et al. 2014, Nugraha and Sugardjito 2009 |
|  | |  | |  |
| Relocation of people | | People relocated (by force or voluntarily) in order to reduce conflict occurrence | | Evaluated; Harihar et al. 2014 Recommended; Nyhus and Tilson 2004, Dunham et al. 2010 |
| Reporting of incident | Data collection of incidents and extent of conflict by organized party | | Evaluated; Tweheyo et al. 2012 | |
| Response teams | Local or government run teams of trained individuals to respond to reports of conflict with either livestock of humans | | Recommended; Inskip et al. 2014, Dhanwatey et al. 2013 | |
| Use of technology | Use of portable technology to monitor, report, and document conflict | | Evaluated; Barlow et al. 2010 Recommended; Farhadinia et al. 2014, Neto et al. 2011 | |
|  |  | |  | |
